# DisProt in 2026: enhancing intrinsically disordered proteins accessibility, deposition, and annotation

**DOI:** 10.1093/nar/gkaf1175

**Published:** 2025-11-17

**Authors:** Maria Victoria Nugnes, Kamel Eddine Adel Bouhraoua, Mehdi Zoubiri, Rita Pancsa, Erzsébet Fichó, DisProt Consortium, Alexander M Monzon, Ana M Melo, Edoardo Salladini, Emanuela Leonardi, Federica Quaglia, Daniyal Nasiribavil, Hamidreza Ghafouri, Gobeill Julien, Emilie Pasche, Patrick Ruch, Paul Van Rijen, László Dobson, Marco Schiavina, Trinidad Cordero, Zsófia E Kálmán, Ximena Castro, Valentín Iglesias, István Reményi, Mahta Mehdiabadi, Gábor Erdős, Zsuzsanna Dosztányi, Peter Tompa, Damiano Piovesan, Silvio C E Tosatto, Maria Cristina Aspromonte

**Affiliations:** Department of Biomedical Sciences, University of Padova, Padova 35131, Italy; Department of Biomedical Sciences, University of Padova, Padova 35131, Italy; Department of Biomedical Sciences, University of Padova, Padova 35131, Italy; Institute of Molecular Life Sciences, HUN-REN Research Centre for Natural Sciences, Budapest 1117, Hungary; Cytocast Hungary Kft, Budapest 1052, Hungary; Institute of Molecular Life Sciences, HUN-REN Research Centre for Natural Sciences, Budapest 1117, Hungary; Department of Biomedical Sciences, University of Padova, Padova 35131, Italy; Department of Biomedical Sciences, University of Padova, Padova 35131, Italy; Institute of Biomembranes, Bioenergetics and Molecular Biotechnologies, National Research Council (CNR-IBIOM), Bari 70126, Italy; Department of Biomedical Sciences, University of Padova, Padova 35131, Italy

## Abstract

DisProt (https://disprot.org/) is an open database integrating experimental evidence on intrinsically disordered proteins (IDPs), intrinsically disordered regions (IDRs), and their functions. Over the past two years, the database has grown over 20%, now comprising 3201 IDPs and 13 347 pieces of evidence, including over 1500 new structural state annotations and >1300 new function annotations. DisProt has systematically adopted the Minimum Information About Disorder Experiments (MIADE) guidelines, more than doubling annotations with experimental details and improving the interpretability of disorder-related experiments. The website has evolved into a hybrid knowledgebase and deposition system, introducing a Deposition Page that allows direct submissions by external users. Through BLAST-based homology propagation in MobiDB, DisProt disorder regions and linear interacting peptides have been extended from hundreds to hundreds of thousands of proteins across >11 000 organisms. This new release marks a paradigm shift by integrating computational predictions as valid evidence and introducing major updates and restructuring of the IDP Ontology, enhancing accuracy, interoperability, and semantic clarity. DisProt continues to support community engagement through training resources together with DisTriage, an AI-based literature triage tool, providing curators with regularly updated lists of prioritized publications.

## Graphical Abstract

**Graphical Abstract:**
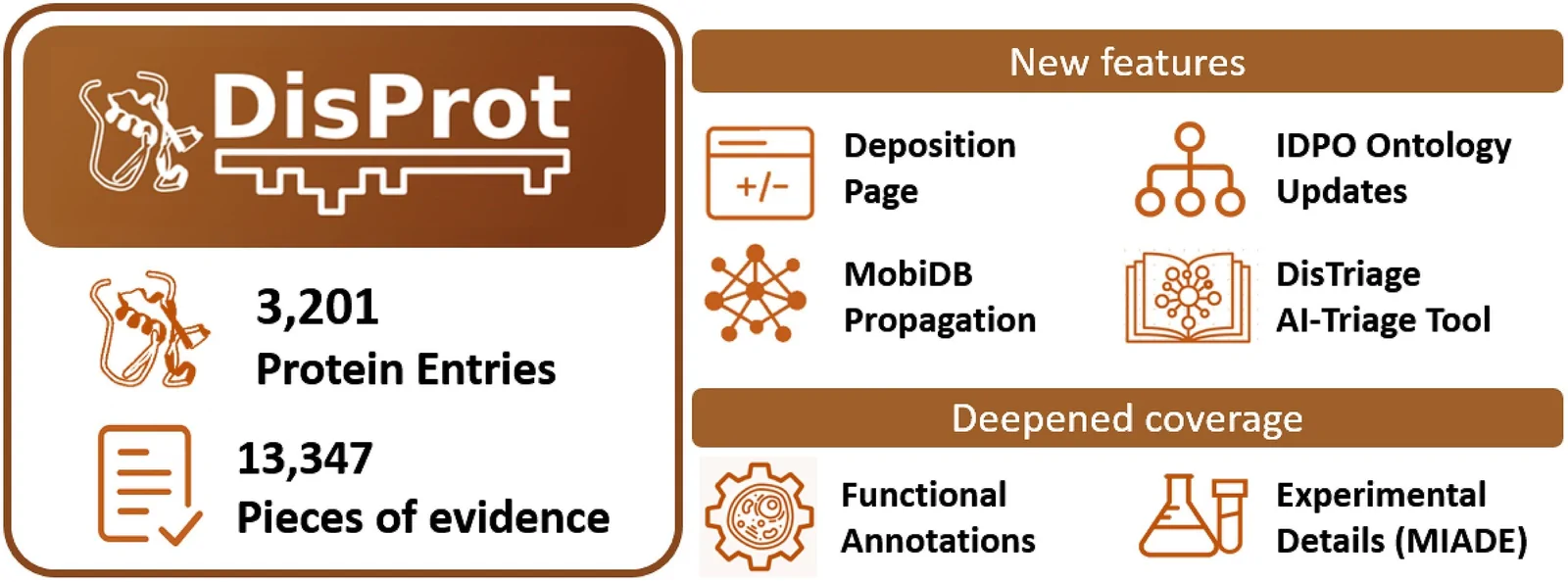

## Introduction

Understanding protein function is at the forefront of contemporary biological research, as it provides insights for outlining biology at the molecular level, developing novel biotechnological tools, and targeting disease. For almost a century, these studies relied on the structure–function paradigm, which stated that a well-defined three-dimensional structure is the prerequisite of protein function. The spectacular success of this paradigm is witnessed by >240 000 high-resolution structures in the Protein Data Bank (PDB) [1] and a dozen or so Nobel prizes given for solving and interpreting protein structures. As an exception to this rule, it became increasingly common that many proteins or regions of proteins lack stable 3D structures but assume rapidly interconverting, dynamic ensembles of conformations, i.e. are intrinsically disordered proteins (IDPs) [2]. The integration of experimental techniques with advanced computational methods fosters a more comprehensive understanding of these elusive systems [3]. As this phenomenon is prevalent in the proteome and such proteins play important signaling and regulatory roles and are also frequently involved in diseases [4], the concept of structural disorder has become central in our conceptualization of molecular cell biology; this field overlaps with proteins that lack recognizable structural homologues in the proteome, i.e. the “dark proteome” [5]. Conclusive evidence for structural disorder now comes from a broad range of direct and indirect experimental observations, both *in vitro* and *in vivo* [6], and also a range of functional and evolutionary considerations and computational modeling [2, 7] such as AlphaFold [8] and RoseTTAFold [9]. The importance of the field is underlined by the fact that structural disorder is prevalent in the proteome [10], with many IDPs playing central roles in key cellular regulatory pathways, including transcription, signaling, cytoskeleton organization, cell cycle control, receptor regulation, biomineralization, and chaperone functions [2, 7]. Recent impetus of the field is generated by the observation that IDPs [or proteins with intrinsically disordered regions (IDRs)] participate in the formation of biomolecular condensates via liquid–liquid phase separation [11], opening potential avenues for drug development [4, 11, 12]. Given these advancements, the study of IDPs has transitioned into a foundational concept in molecular and cellular biology. Research combines experimental and computational approaches to characterize structural ensembles and explore potential drug targets [4, 13, 14], as well as predicting intrinsic disorder, disordered binding regions, and even the conformational ensembles of IDPs/IDRs [3, 15]. As recently the whole computational field—including studies of IDPs—is in a transition to developing and applying large machine learning models [16, 17], there is an increasing need for databases presenting solid data on structural disorder and/or disorder-related functional annotations. Indeed, there are several databases of disorder-related information at different levels of resolution, such as MobiDB [18], with full coverage of about 245M computational disorder predictions; Protein Ensemble Database (PED) [19], with ~700 conformational ensembles describing 300 IDPs and IDRs; and the Eukaryotic Linear Motif (ELM) database, which is the repository of ~4300 instances of functionally validated short linear motifs (SliMs), often falling into disordered regions [20]. However, DisProt is the primary database of experimentally validated disorder annotations. It started in 2005 with 100 IDP sequences [21] and grew to 2600 proteins by its latest edition DisProt 9 in 2024 [22]. It is now integrated with major resources such as UniProtKB [23], PDBe [24], and Gene Ontology [25] and contributes as the reference dataset for the Critical Assessment of Protein Intrinsic Disorder prediction (CAID) experiment [26, 27], aimed at benchmarking relevant ID and binding predictors.

Compared to the version published in 2024, DisProt has improved its data annotation, currently including over 3000 protein entries and over 13 000 pieces of evidence. When considering the number of disorder evidences following the Minimum Information About Disordered Experiments (MIADE) guidelines [6], the growth is more than doubled (from 1028 to 2359). Thematic datasets have also increased from five to nine, introducing four new categories. Recently, DisProt has also implemented its own Intrinsically Disordered Proteins Ontology (IDPO) and adopted three new Evidence and Conclusion Ontology (ECO) terms [28]. When considering technical improvements, DisProt integrated a new feature, the Article Deposition Page, and provides an automated publication triage tool (DisTriage). The curator community is further supported through training initiatives (such as ELIXIR courses and expert-led sessions), curation guidelines, and formal recognition via APICURON [29]. In the following sections, we provide a detailed overview of the advancements introduced in DisProt version 9.8.

## DisProt in 2026: progress and new features

### New feature: DisProt article deposition page

**Figure 1.**
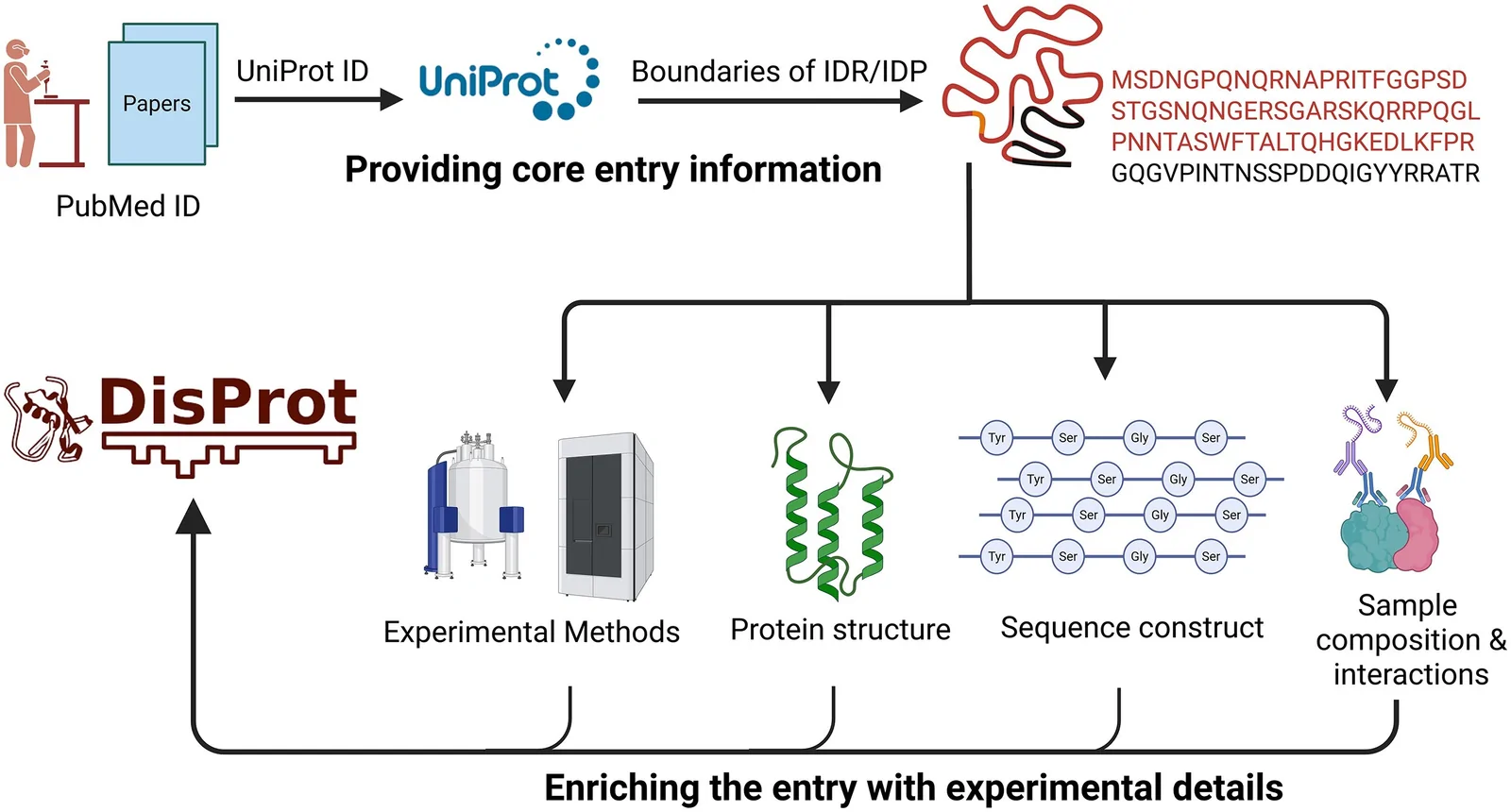
Workflow describing the important steps in the article deposition process. Created in BioRender. Aspromonte, M. (2025) https://BioRender.com/z5rq1gc.

We continued to develop and expand the DisProt database, with a focus on community engagement and data transparency. A major advancement introduced in 2025 is a dedicated Article Deposition Page (https://deposition.disprot.org/), which allows users to contribute directly by scientific publications that contain experimental evidence on IDPs/IDRs and their functions. Contributors should provide the reference ID (DOI, PubMed ID) to their article, whether already published or under review, along with information needed to describe the IDPs/IDRs characterized, following the DisProt curation guidelines [30]. This new functionality revolutionizes DisProt, turning it into a hybrid resource that serves both as a knowledgebase and as a deposition platform, in compliance with ELIXIR Core Data Resource recommendations (https://elixir-europe.org/platforms/data/core-data-resources). The interface is accessible on phone and tablet screens and guides contributors through the structured submission of disordered state data. By enabling direct user submissions, DisProt expands its mission from annotation to active data collection. Deposition focuses on reporting publications that provide experimental evidence of disorder. Users are guided through a short, structured form that collects the essential publication details and the key experimental observations described in the article (Fig. 1). All deposited data are reviewed by expert curators before publication to maintain scientific rigor and high-quality standards. The design of the deposition page is clean and responsive with a modular structure that guides users step-by-step through the submission process. It is intended for results that have been published or are under review as part of peer-reviewed papers. Two distinct workflows are available, “*Complete*” and “*Partial*”submissions, where the first one includes both basic and additional experimental details. Before starting, submitters are required to provide email contact, ORCID ID and submission minimum information: (i) DOI or PubMed ID of the reference; (ii) UniProt ID and organism; (iii) region boundaries corresponding to the IDP/IDR described in the article and/or functions; and (iv) experimental method used to detect disorder (e.g. NMR, SAXS, CD). In addition, it is recommended to report experimental details, as described in the MIADE guidelines, as well as relevant database cross-references (e.g. PDB, BMRB, SASBDB) [24, 31, 32]. The deposition system includes an integrated smart suggestion engine to help the user.

### Protein coverage, re-annotation, and functional enrichment

Version 9.8 of DisProt (released in June 2025) contains 3201 protein entries and 13 347 pieces of evidence including disorder structural state, transition, and function annotated with IDPO and GO terms. Table 1 provides the absolute counts of proteins and annotations for each IDPO and GO aspect, with a 20.8% and 22.33% growths, respectively, since the last publication in 2023 [22].

**Table 1.** Functional and structural annotations in DisProt for each main ontology branch

Ontology	Aspect	Proteins	Annotations
IDPO	Structural state	3195	7381
IDPO	Structural transition	582	962
IDPO	Disorder function	642	1022
GO	Molecular function	1109	3394
GO	Biological process	253	530
GO	Cellular component	34	58

The total number of proteins and annotations for each IDPO aspect (structural state, structural transition, and disorder function) and GO domain (molecular function, biological process, and cellular component) in DisProt 9.8 is shown.

Importantly, systematic re-annotation and quality refinement were carried out on 999 pieces of evidence. This effort included the designation of obsolete annotations (528 pieces of evidence) when supporting data were no longer reliable, adjustment of region boundaries to align with experimental observations, addition of experimental MIADE details [6], and replacement of generic GO and IDPO terms with more precise ontology descriptors. For example, in DP00116r003 ion binding (GO:0043167) was replaced by calcium ion binding (GO:0005509). Over 2500 annotations underwent quality control checks to improve data reliability, raising the total validated content to 52%. Together, these efforts reflect the emphasis on more accurate, consistent, and interpretable biocuration. This expansion has been significantly supported by community contributions: ~19% of new entries and 46.5% of new annotations were curated by external contributors. These figures highlight the success of our international outreach and training programs in fostering a collaborative biocuration environment.

**Figure 2.**
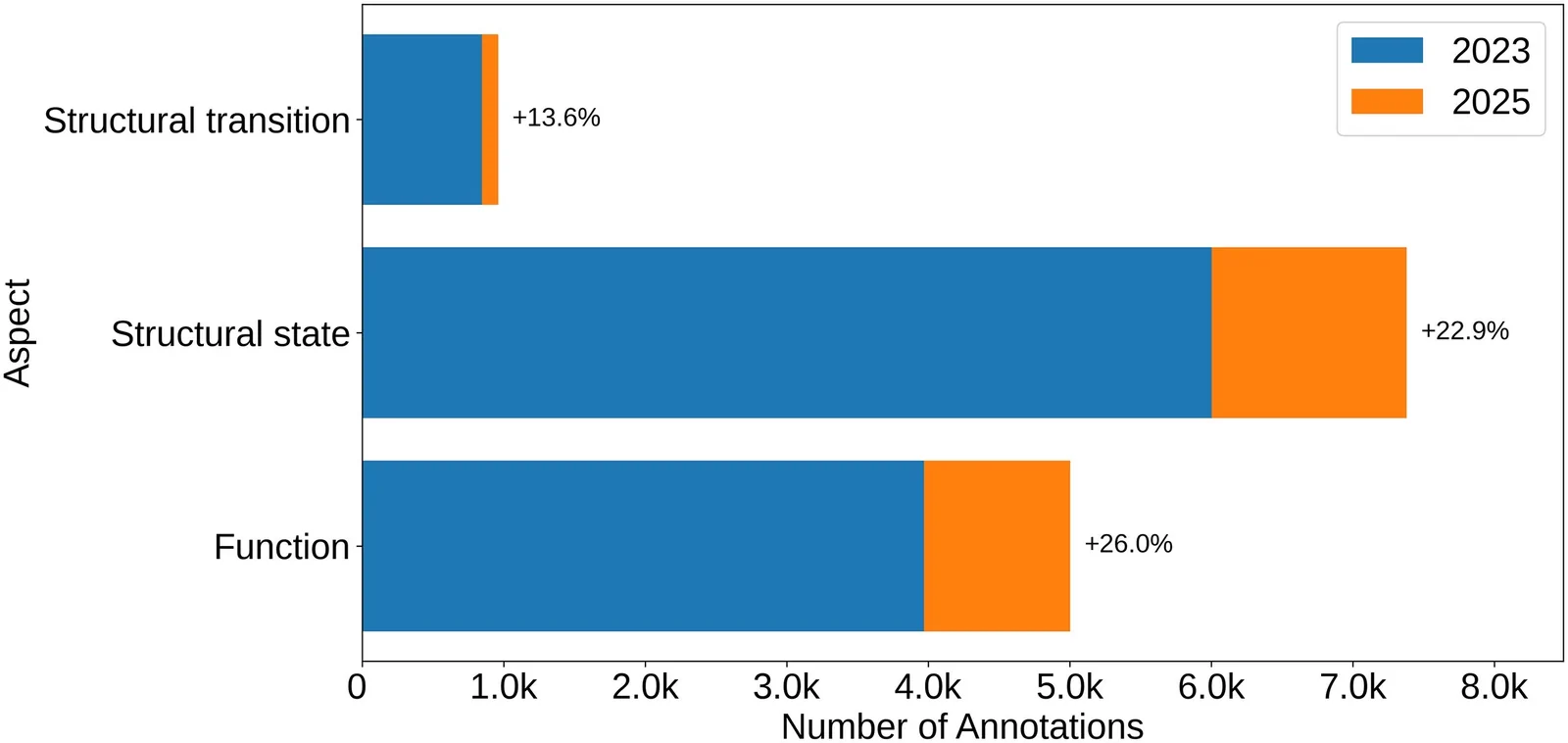
Comparison of the number of annotations between 2023 and 2025 across different aspects: Structural state, Structural transition, and Function. The *Function* category aggregates all disorder-related functional annotations, combining IDPO Disorder Function terms with Gene Ontology (GO) Molecular Function, Biological Process, and Cellular Component annotations. Percentages next to each bar indicate the relative increase in annotations compared with 2023.

During these last two years, the expansion of IDP/IDR functional annotations was one of the main focuses of the curation efforts. Functional annotations increased by 26%, while structural state annotations grew by 22.9% and structural transitions by 13.6% (Fig. 2). This effort resulted in a total of 3982 GO annotations—an increment of 23% since the last count. The functional landscape illustrates this growth in detail.

**Figure 3.**
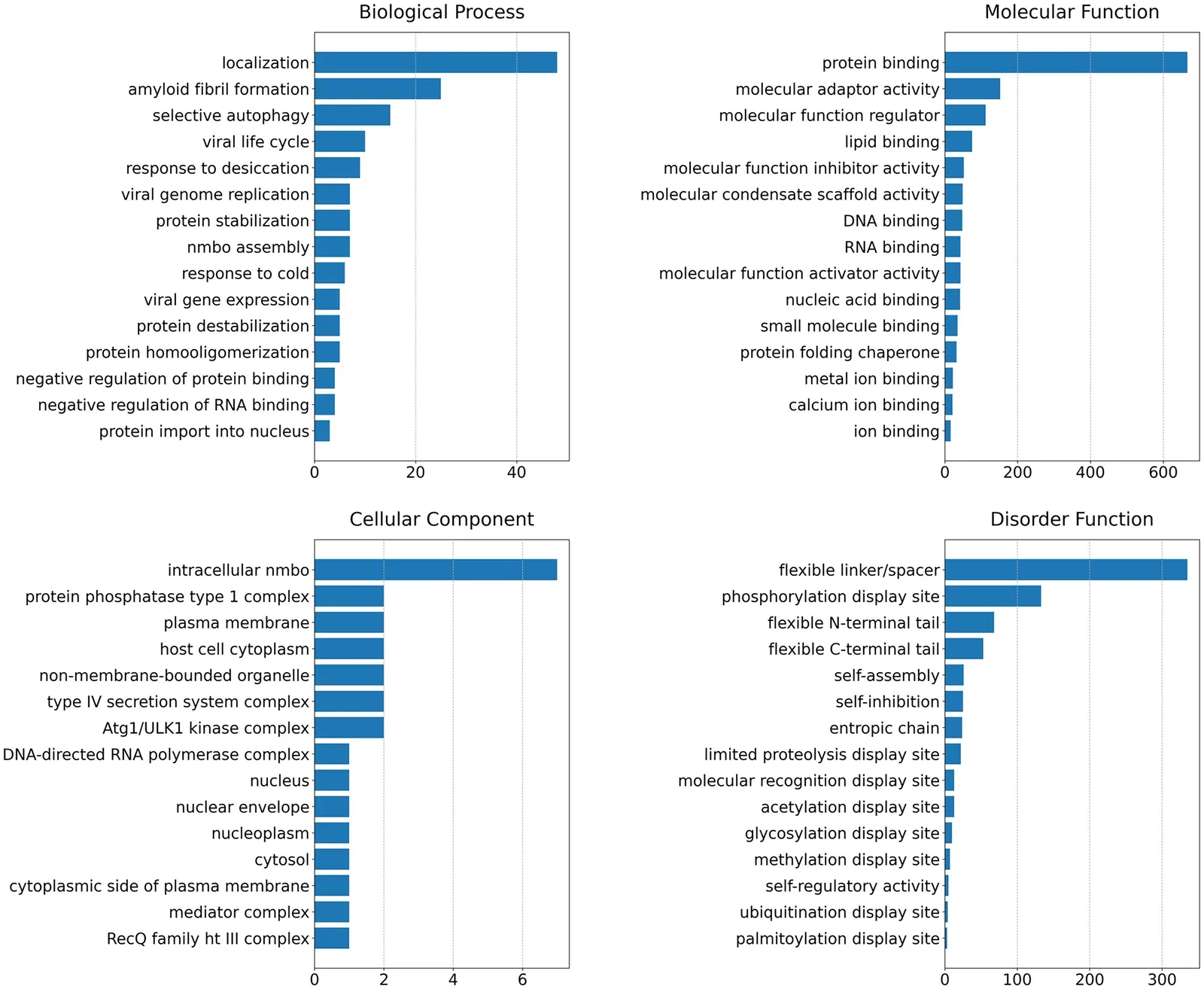
Distribution of the 15 most frequently used GO and IDPO functional terms curated in DisProt. The plot illustrates both the number of proteins annotated with each term and the diversity of terms within each functional category. Proteins with multiple identical annotations, e.g. when different articles report the same experimental evidence, were counted only once. Abbreviations: non-membrane-bounded organelle (nmbo); helicase-topoisomerase (ht).

Among GO terms, protein binding (GO:0005515) dominates the Molecular Function category, with >600 annotations, highlighting the central role of IDRs as hubs of interaction in cellular networks. Structural plasticity underlies this enrichment and also explains the high frequency of molecular adaptor activity (GO:0060090), the second most common term within this class. Annotations are more diverse for Biological Processes, with localization (GO:0051179) being the most represented, followed by amyloid fibril formation (GO:1990000), reflecting the dual role of IDPs in normal physiology and in pathological aggregation. Although Cellular Component annotations are comparatively sparse, they highlight the contexts in which IDPs operate, including intracellular non-membrane-bounded organelles (GO:0043231) and protein complexes. In-depth insights into DisProt data are provided in Fig. 3.

Over 300 new IDPO annotations (+39%) were used to describe disorder-related functions. The most frequent are flexible linkers/spacers (IDPO:0000033) and phosphorylation display sites (IDPO:0000045), consistent with the description of IDRs as flexible connectors and targets of regulatory modifications.

### Expanded integration of MIADE terms for experimental precision

Since 2023, DisProt has systematically adopted the MIADE guidelines [6] to improve the description and interpretability of the experimental evidence. MIADE specifies the key elements required to draw unambiguous conclusions from disorder-related experiments. These include c*onstruct alterations*, encoding deviations from the wild-type UniProt-defined protein sequence (e.g. tags, PTMs, mutations); *experimental components*, elements present in the experiment such as cofactors, ligands, binding partners, or other molecules influencing the measurement; and *experimental conditions*, i.e. parameters that can affect the biological interpretation of an experimental observation. Since the last publication, the number of MIADE-annotated evidence items has more than doubled (from 1028 to 2359), giving DisProt a much more detailed view of experimental setups.

**Figure 4.**
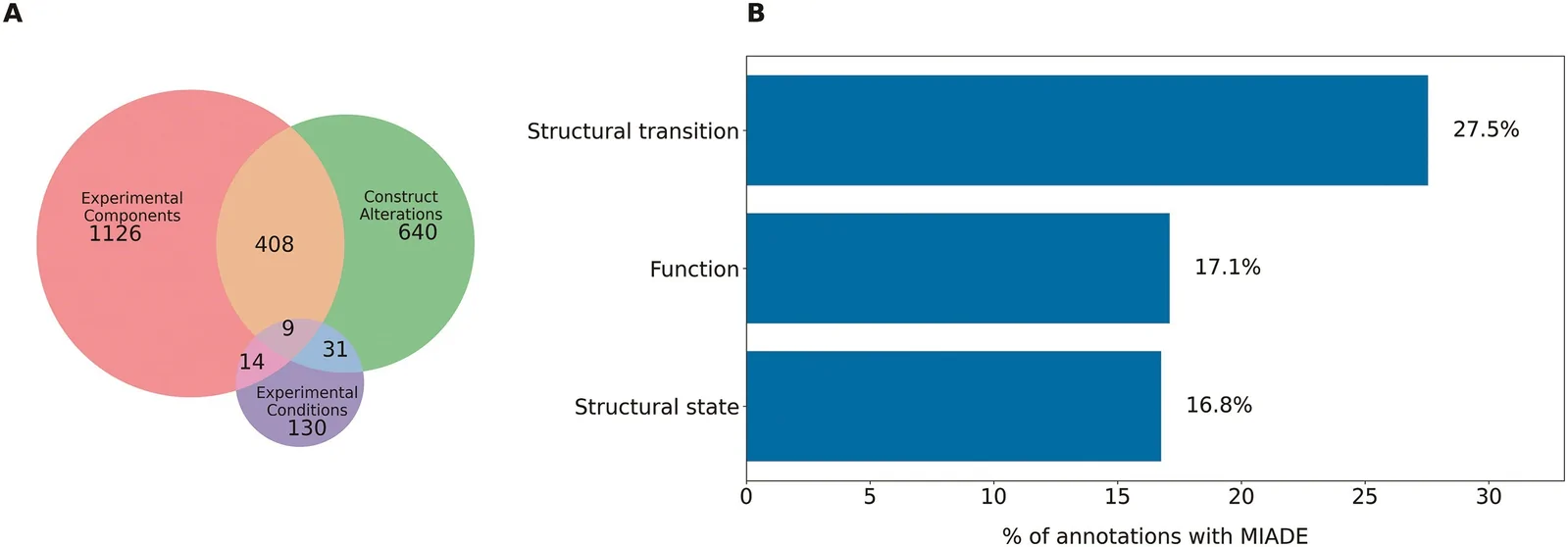
Overview of MIADE information coverage in DisProt annotations, highlighting distribution across different annotation types and experimental details. (**A**) The Venn diagram reports DisProt annotations (version 9.8) containing MIADE information among the three different categories. (**B**) Proportion of annotations with MIADE descriptors by annotation type. Structural transitions show the highest density of experimental details (27.5%), followed by Function annotations (17%) and Structural state annotations (16.8%).

Notably, a single piece of evidence can contain multiple MIADE descriptors. For example, it may report both construct alterations and experimental components, reflecting the multidimensional context of disorder experiments (Fig. 4A). The Venn diagram illustrates these overlaps, e.g. 408 annotations combine experimental components and construct alterations, while 9 annotations include all three category descriptors, thus offering a particularly detailed description of the experimental setup.

The impact of MIADE is most evident in structural transitions, where >27% of all transition annotations include experimental details. In comparison, ~17% of functional annotations and 16.8% of structural state annotations contain MIADE descriptors (Fig. 4B). This higher frequency reflects the strong dependence of structural transitions on experimental setups, making contextual details essential for interpretation. For instance, interacting proteins—reported in DisProt under *experimental components—* can influence the structural state of disordered regions. For example, paratox protein from *Streptococcus pyogenes* serotype M3 (UniProtKB:A0A0H2UWN8) undergoes folding-upon-binding when interacting with the signal receptor and transcription factor ComR (UniProtKB:Q8DWI6), as captured in DisProt in DP04243r004 [33].

### Integrating annotation across the tree of life

**Figure 5.**
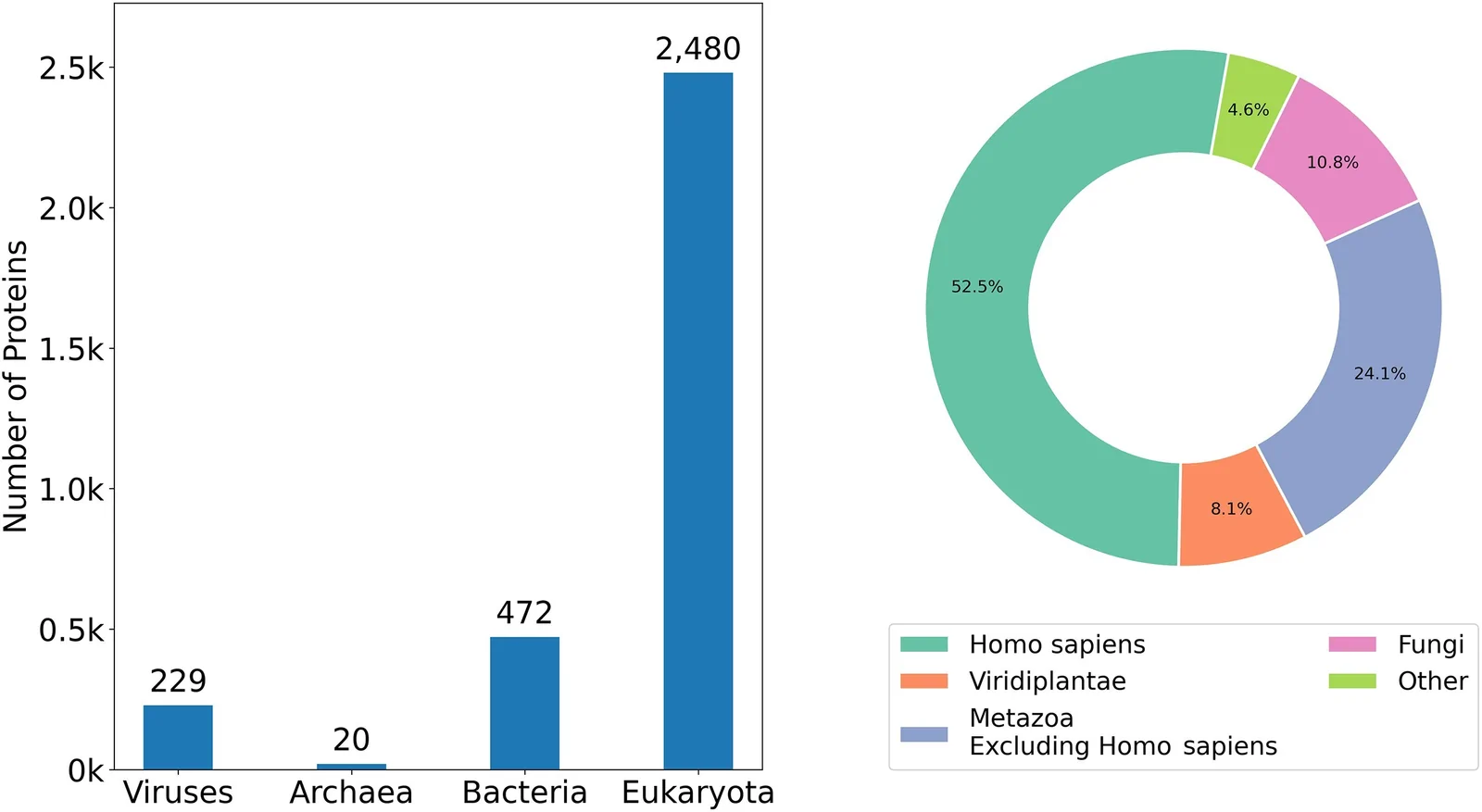
Taxonomic distribution of annotated proteins in DisProt. (**A**) Number of proteins annotated across the four major domains of life. (**B**) Number of proteins annotated in different eukaryotic species.

The current DisProt release features extensive updates not only for human proteins but also across a wide range of organisms spanning the four major domains of life: viruses, archaea, bacteria, and eukaryota. The total number of proteins collected in DisProt in all major domains of life, as well as the percentage of species among eukaryota, is reported in Fig. 5A and B, respectively. Eukaryota represent the predominant group, followed by bacteria. Although bacterial entries remain fewer than eukaryotic ones, their increase marks an important step toward a more comprehensive coverage of disorder across taxa.

Thematic datasets have also been enriched, increasing from five to nine since the 2023 update, focusing on specific biological functions, interactions with other biomolecules (e.g. RNA), or involvement in pathophysiological conditions. These collections further contribute to expanding the representation of proteins across different organisms (Table 2), e.g. inclusion of bacterial virulence proteins.

**Table 2.** Number of IDPs and IDRs included in the thematic datasets

Dataset	Proteins	Annotations	GO Annotations	IDPO Annotations	Average Disorder Content (%)
Condensate-related proteins	182	1511	638	873	39
RNA-binding proteins	213	1360	493	867	25.38
Age-related disorders proteins	42	256	90	158	26
Bacterial virulence-related proteins	76	337	80	257	15.25

For each dataset, GO and IDPO annotations are calculated at the level of annotated regions, while the disorder content is computed at the protein level by averaging the percentage of disorder content across all proteins in the unique thematic dataset.

The newly added thematic dataset, Bacterial virulence-related proteins, contains proteins that are required for or contribute to bacterial pathogenicity. It highlights the role of intrinsic disorder in host–pathogen interaction mechanisms, immune evasion, and activation of toxic functions. Disordered regions often provide the conformational flexibility required for molecular recognition, post-translational regulation, and environmental responsiveness, making them particularly suited to modulate complex infection strategies.

For example, cholera enterotoxin subunit A (DisProt:DP00250; UniProtKB:P01555) is part of the heterohexameric AB5 complex. This potent bacterial enterotoxin is responsible for the massive fluid secretion observed during infection with *Vibrio cholerae* [34]. This protein contains IDRs [35] that are functionally critical for toxin activation. Specifically, the IDR (residues 19–212) interacts with protein disulfide isomerase in the lumen of the endoplasmic reticulum, facilitating disassembly and unfolding of the toxin once its A chain has been cleaved [36].

### Homology propagation and MobiDB integration

Beyond the manually curated entries described above, the taxonomic coverage of DisProt has been further expanded by integrating its annotations into MobiDB [18, 37] and propagating them through a homology-based procedure that uses BLAST [38] to assess sequence similarity against the entire UniProtKB database. In this approach, DisProt proteins are aligned against the full UniProtKB sequences, and manually curated regions are projected into proteins based on stringent similarity criteria evaluated at the region level. Region homology is inferred by filtering BLAST high-scoring-pair alignments with a maximum *E*-value <0.01, an alignment length of at least 10 residues, coverage (i.e. sequence overlap) of 90% for both sequences, and a minimum sequence identity of 80% with at most 20% gaps. All these parameters are calculated after aligning the entire DisProt sequences against the UniProtKB database and after trimming the alignments for the corresponding ID region(s) in the query sequence [37]. This strategy has enabled large-scale expansion of annotations beyond the manually curated DisProt entries, ensuring that functional disorder information reaches a much broader set of proteins. Starting from the DisProt seed, the number of proteins that got a homology annotation are 330 748 and those that inherited a linear interacting peptide (LIP) annotation are 100 078. This expands the initial DisProt dataset by two orders of magnitude. The impact of the MobiDB-driven annotation expansion is also evident in the increased taxonomic diversity of annotated proteins, covering 11 655 different organisms compared with the 447 currently available in DisProt.

When experimental evidence in different homologs is only partial and available for regions located in different positions of the sequence, they are integrated to complement each other at the family level. For example, human Epsin-1 (DisProt:DP02930; UniProtKB:Q9Y6I3) has experimental evidence only for the N-terminal region 1–18 in DisProt. In MobiDB, instead, disorder annotation is extended to the C-terminal region 144–576 from the manually curated evidence in the *Rattus norvegicus* homolog (DisProt:DP00251; UniProtKB:O88339).

DisProt expanded annotations are available in MobiDB in two different datasets, one for disorder only and the other capturing LIP annotations. They can be downloaded from the MobiDB browse page by applying the filter “Feature exist” and the value “homology-disorder-disprot” or “homology-disorder-lip”, respectively.

### Updates to the ontologies contents and structure: ECO and IDPO

This version represents a major paradigm shift for DisProt. Until now, the DisProt curator community has relied exclusively on experimentally validated evidence to support annotations of IDP/IDR structural state. For the first time, computational predictions are also considered a valid source of evidence if manually checked. This change is supported by the clear progress observed in the CAID, which has demonstrated—across three different editions [26, 27, 39]—substantial and consistent improvements in disorder prediction accuracy and reliability. To enable this transition, three new terms have been added into the ECO and integrated in DisProt: intrinsic disorder prediction evidence used in manual assertion (ECO:0008033), author inference based on intrinsic disorder prediction used in manual assertion (ECO:0008035), and curator inference based on intrinsic disorder prediction used in manual assertion (ECO:0008037).

The IDPO (https://disprot.org/ontology), previously used to capture the Structural state, Structural transition and Disorder function of IDPs/IDRs, has undergone major updates to better reflect the current knowledge of IDPs and to ensure compatibility with life sciences standards and formal ontology formats. IDPO has been submitted to the Open Biological and Biomedical Ontology (OBO) Foundry (https://obofoundry.org/about-OBO-Foundry.html). New terms have been added to capture concepts previously missing from the ontology (https://github.com/BioComputingUP/idpo), and its structure has been reorganized to increase interoperability with other biomedical ontologies and enhance semantic clarity in annotation. For example, new terms describe the transition into two separate liquid phases (liquid–liquid phase separation; IDPO:0000025) and the transition from a disordered liquid state into a glass-like state (crystallization; IDPO:0000028). IDPO now also covers functional aspects of IDPs, including the six SLiM site categories used in the ELM database [] (cleavage, degron, etc.) and more functions related to the condensate formation (for example condensate assembler; IDPO:0000061).

### Supporting the community

Over the past two years, the DisProt team has coordinated and supported both users and the curator community, providing training courses and material on the database and curation guidelines [30]. All the training materials are available on the ELIXIR E-learning site (https://elixir.mf.uni-lj.si/) in both English and Spanish, ensuring wider accessibility for an international audience. As part of ongoing efforts, we collaborated with experts on the most common experimental techniques for IDPs, i.e. nuclear magnetic resonance (NMR), small-angle X-ray scattering (SAXS), and single-molecule fluorescence resonance energy transfer (smFRET), to present three specialized training sessions (https://elixir.mf.uni-lj.si/). This initiative aimed to train volunteer curators and researchers and enhance the quality and consistency of annotations while fostering a growing community of contributors.

The DisProt team provides several ways for users to learn more about both retrieving and curating data. We also publish regular updates (every 6 months) on the DisProt web resource (https://disprot.org/release-notes), publishing the new thematic dataset including new entries and a brief description in a blogp ost (e.g. last thematic dataset “Bacterial virulence-related proteins” (https://biocomputingup.github.io/2025/07/07/release_2025_06/) and bi-monthly meeting where users and curators can attend for new announcements, current activities, and future directions.

In support of these efforts, DisProt integrates APICURON, a dedicated platform designed to acknowledge the contributions of biocurators [29]. This integration allows us to track and record the work of both expert and volunteer curators, who are rewarded with badges and medals. Badges are awarded when curators reach milestones such as completing functional annotations or curating experimental details according to MIADE, while medals acknowledge top-performing curators annually and for long-term achievements.

Since 2021, DisProt has served as a reference resource for the CAID, a community-wide initiative that benchmarks the performance of computational methods for IDR and functional site prediction. Now in its third edition, CAID further consolidates the role of DisProt as a central hub for the IDP field, fostering collaboration between experimental and computational communities and driving the development of novel predictive approaches.

### DisTriage: query independent triage as a digest to support AI-readiness

In addition to these improvements, the literature triage strategy has also significantly progressed. Since the DisProt in 2020 [41], the publication triage support services—originally based on estimating the density of descriptors related to IDPs—have been completely refactored with the delivery of the DisTriage (https://biodiversitypmc.sibils.org/distriage) application. This system applies pre-trained language models to automatically rank published articles according to their relevance for disorder-related protein curation. Specifically, we fine-tuned a bidirectional model (PubMedBERT-base-uncased-abstract-fulltext) for binary classification of biomedical articles into positive and negative/far-negative relevance classes. The resulting model is geared toward supporting high-recall document triage for downstream text ranking APIs. The demonstrator is based on the triage infrastructure of the SIB Text Mining, which operates triage services for ELIXIR/SIB Core Data Resources, such as the CelloSaurus [42].

To construct the training dataset, positive and negative PMIDs (*N* = 500) were combined with far-negative examples randomly sampled from the MEDLINE corpus. Abstracts were retrieved from MEDLINE and full texts from PMC using the SIBiLS [43] Fetch API, giving preference to PMC full texts. Each sample consisted of the article title and body text (full text or abstract), truncated to a maximum length to ensure consistency. Crucially, *DisTriage* is now delivered as a weekly report, providing curators with regularly updated lists of prioritized publications.

We manually evaluated the precision of the system by measuring the fraction of true positive articles at different ranks (10, 20, and 50) as shown in Table 3. Fine-tuned as a ranker (i.e. a system that ranks results by relevance), DisTriage is able to achieve relatively good precision at high ranks (70%), but the discriminative power degrades when considering the top 50 results returned by the system.

**Table 3.** True positives and precision of DisTriage for top 10, top 20, and top 50 ranked articles

	True positive instances	Precision (%)
Top 10	7	70
Top 20	13	65
Top 50	27	54

This continuous and automated provision of candidate literature streamlines the identification of relevant studies and ensures that DisProt curation keeps pace with the rapid growth of protein disorder research. The system has room for improvement thanks to the continuous monitoring of the literature and the curation workflow of the resource. Unlike most literature-based curation support pipelines, the system records not only the acquisition of positive (i.e. articles judged as relevant to curate DisProt) but also—and as importantly for the sake of triage—of negative instances (i.e. articles judged as irrelevant and usually ignored by databases). Such incremental improvements have little impact on the curation workflows, whereas they significantly improve the AI-readiness of the database.

## Conclusion

DisProt is a comprehensive database that both collects curated experimental evidence and provides a framework for harmonizing how IDPs are described and interpreted across studies. The latest DisProt release marks a major step forward in the standardization and integration of knowledge on IDPs/IDRs. Beyond the quantitative expansion of its content (increased by 20.8% in terms of proteins and by 23% in terms of evidence items), the enrichment of entries with detailed functional annotations and experimentally supported information (MIADE-annotated evidence entries have more than doubled) greatly enhances the biological depth and reliability of the database. Through the integration of DisProt annotations in MobiDB and their propagation across homologous proteins using stringent sequence similarity criteria, functional disorder information now reaches a far broader taxonomic spectrum, significantly extending the impact of curated data annotated in DisProt.

Another key contribution of this last DisProt version is the update of IDPO, which now provides a more accurate conceptual framework for describing structural and functional features of IDPs/IDRs. This revision represents a crucial step for the entire IDP community, supporting a shared framework and fostering greater standardization in the annotation and interpretation of disordered proteins. This update also lays the foundation for future work, including formalizing a logically well-formed and scientifically accurate ontology by OBO Foundry and the expansion of IDPO to capture all aspects of protein disorder. The revised ontology also captures additional functional aspects, including linear motifs (ELMs), thereby extending standardization efforts toward the annotation of short, functional sequence elements. Additionally, the adoption of new ECO terms for describing and collecting disorder prediction evidence not only underscores the growing importance of CAID results but also represents a significant improvement for collecting evidence on disorder function even when experimental structural information is lacking. This scenario is becoming increasingly frequent, given the reliability of current disorder predictors. Alongside this expansion, the DisProt platform has undergone significant technical and conceptual advancements, with a strong focus on community engagement and data transparency. A dedicated Deposition Page (https://deposition.disprot.org/) now enables external users to submit IDR/IDP experimental evidence in a standardized format aligned with DisProt curation guidelines. In addition, the DisTriage application was released: a system that employs pre-trained language models to automatically rank published articles according to their relevance for protein disorder curation. Together, these developments consolidate DisProt’s role as the cutting-edge reference resource for IDP research. Its integration into ELIXIR core data resources, such as InterPro [44] and UniProtKB, further ensures its accessibility, interoperability, and long-term sustainability for the protein disorder community.

## Data Availability

The data that support the findings of this study are openly available in DisProt at https://disprot.org/.

## Appendix

### DisProt Consortium

Alexander M. Monzon^1^, Ana M. Melo^5,6^, Edoardo Salladini^7^, Emanuela Leonardi^1^, Federica Quaglia^1^, Daniyal Nasiribavil^1^, Hamidreza Ghafouri^1^, Gobeill Julien^8,9^, Emilie Pasche^8,9^, Patrick Ruch^8,9^, Paul Van Rijen^8,9^, László Dobson^2,10^, Marco Schiavina^11^, Trinidad Cordero^1^, Zsófia E. Kálmán^12^, Ximena Castro^1^, Valentín Iglesias^13^, István Reményi^14^, Mahta Mehdiabadi^1^, Gábor Erdős^15^, Zsuzsanna Dosztányi^15^.

5. iBB—Institute for Bioengineering and Biosciences, Instituto Superior Técnico, Universidade de Lisboa, Lisboa 1049-001, Portugal

6. Associate Laboratory i4HB—Institute for Health and Bioeconomy at Instituto Superior Técnico, Universidade de Lisboa, Lisboa 1049-001, Portugal

7. Department of Drug Science and Technology, University of Turin, Turin 10125, Italy

8. Swiss Institute of Bioinformatics, Geneva 1206, Switzerland

9. BiTeM Group, Information Sciences, HES-SO/HEG Geneva, Carouge 1227, Switzerland

10. Department of Bioinformatics, Semmelweis University, Budapest 1094, Hungary

11. Magnetic Resonance Center and Department of Chemistry "Ugo Schiff", University of Florence, Florence 50019, Italy

12. Faculty of Information Technology and Bionics, Pázmány Péter Catholic University, Budapest 1083, Hungary

13. Clinical Research Centre, Medical University of Białystok, Kilińskiego 1, Białystok 15-369, Poland

14. Department of Artificial Intelligence, Faculty of Informatics, ELTE Eötvös Loránd University, Budapest 1117, Hungary

15. Department of Biochemistry, Eötvös Loránd University, Budapest 1117, Hungary
