# Correction to ‘DisProt in 2026: enhancing intrinsically disordered proteins accessibility, deposition, and annotation’

**DOI:** 10.1093/nar/gkag1124

**Published:** 2026-10-24

This is a correction to: Maria Victoria Nugnes, Kamel Eddine Adel Bouhraoua, Mehdi Zoubiri, Rita Pancsa, Erzsébet Fichó, DisProt Consortium, Peter Tompa, Damiano Piovesan, Silvio C E Tosatto, Maria Cristina Aspromonte, DisProt in 2026: enhancing intrinsically disordered proteins accessibility, deposition, and annotation, *Nucleic Acids Research*, Volume 54, Issue D1, 6 January 2026, Pages D383–D392, https://doi.org/10.1093/nar/gkaf1175

The following change has been made to the originally published paper.

The following statement has been added to the Funding section: “Views and opinions expressed are however those of the author(s) only and do not necessarily reflect those of the European Union or the European Research Executive Agency (REA). Neither the European Union nor the granting authority can be held responsible for them.”

